# Subjective and Objective Quality Assessments of Display Products †

**DOI:** 10.3390/e23070814

**Published:** 2021-06-26

**Authors:** Huiqing Zhang, Donghao Li, Yibing Yu, Nan Guo

**Affiliations:** 1 Faculty of Information Technology, Beijing University of Technology, Beijing 100124, China; zhq@bjut.edu.cn (H.Z.); yuyibing@bjut.edu.cn (Y.Y.); guonan03@126.com (N.G.); 2 Engineering Research Center of Digital Community, Ministry of Education, Beijing 100124, China; 3 Beijing Laboratory for Urban Mass Transit, Beijing 100124, China

**Keywords:** display product, no-reference, subjective and objective quality assessment

## Abstract

In recent years, people’s daily lives have become inseparable from a variety of electronic devices, especially mobile phones, which have undoubtedly become necessity in people’s daily lives. In this paper, we are looking for a reliable way to acquire visual quality of the display product so that we can improve the user’s experience with the display product. This paper proposes two major contributions: the first one is the establishment of a new subjective assessment database (DPQAD) of display products’ screen images. Specifically, we invited 57 inexperienced observers to rate 150 screen images showing the display product. At the same time, in order to improve the reliability of screen display quality score, we combined the single stimulation method with the stimulation comparison method to evaluate the newly created display products’ screen images database effectively. The second one is the development of a new no-reference image quality assessment (IQA) metric. For a given image of the display product, first our method extracts 27 features by analyzing the contrast, sharpness, brightness, etc., and then uses the regression module to obtain the visual quality score. Comprehensive experiments show that our method can evaluate natural scene images and screen content images at the same time. Moreover, compared with ten state-of-the-art IQA methods, our method shows obvious superiority on DPQAD.

## 1. Introduction

With the arrival of the era of big data, various terminal electronic devices are needed to support human daily life. For example, distance education, digital entertainment, security monitoring, and even daily chores such as refreshing a microblog or ordering takeout food are no exception. Such a huge demand has led to the increasing popularity and development of terminal electronic devices such as mobile phones, computers, and tablet computers. Therefore, how to obtain the user’s real visual experience of the above-mentioned electronic products and improve the user’s visual experience on this basis has attracted widespread attention from researchers. Note that there is a variety of display products and their screen parameters vary greatly. Display products with different screen parameters bring different visual experiences to users, which greatly affects the user experience. Therefore, it is urgent to evaluate the visual quality of display products and guide the setting of screen parameters of display products according to the user’s visual experience.

In general, we divide the image quality assessment into two different types of methods: subjective quality assessment and objective quality assessment. First of all, subjective quality assessment means that the observer directly gives the quality score of a given image. Specifically, this method involves inviting a large number of testers (namely, testers of different ages, different genders, and different occupations) to evaluate the scores of the images, and then performing data cleaning on the quality score of each image, and finally the average or difference value of the above-mentioned large number of quality scores is used as the final quality score. Nowadays, some researchers expect to adopt crowdsourcing and crowd-workers [1] to realize the subjective image quality assessment. As this method refers to the direct evaluation of the overall quality of the image by the human audience, and the human audience are the ultimate users, so that the subjective quality assessment is decisive, and it is also the final criterion for judging whether the objective quality assessment algorithm is effective. Under normal circumstances, observers use single stimulation method or stimulation comparison method when scoring a given image, but either subjective evaluation method is unstable due to the observer’s viewing conditions, personal preferences, habits and other factors. Therefore, in order to improve the reliability of subjective scoring, we effectively integrate the above two methods and make them supervise each other in this paper, so that the subjective scores of the quality assessment database of display products (DPQAD) are accurate and reliable.

Second, although subjective quality assessment is indispensable, it cannot be widely promoted in practical application because it consumes a lot of manpower, material, and financial resources, and also has disadvantages such as being highly time-consuming and having unstable evaluation results. According to the above situation, reliable, and effective objective quality assessment method has become the subject of many researchers. Objective quality assessment is to design a certain model essentially and make it close to the human visual system. Many objective image quality assessment models which are based on low level vision [2,3,4], brain theory [5,6], contrast enhancement [7,8], and statistics [9] have been proposed. Then, note that although the above objective quality assessment methods have achieved good performance in evaluating natural scene images (NSIs), they are often inferior in evaluating screen content images (SCIs). The reason for this phenomenon is that there is the huge difference between NSIs and SCIs: NSIs have rich colors, thick lines, and complex textures, while SCIs have monotonous colors, thin lines, and simple textures. The above reasons have led to a series of challenging problems. It can be seen from the works in [10,11] that the quality evaluation methods that are applicable to NSIs or SCIs solely, but cannot evaluate the above two different types of images at the same time effectively. Considering the needs of practical applications, display products will not only display NSIs with rich colors and complex textures, but also display SCIs with limited colors and simple shapes. Therefore, the quality assessment of display products is different from existing works, resulting in the existing works cannot be directly applied to the quality assessment of display products. It is also because it is very difficult to evaluate the quality of display products, so there has been very few studies in this area so far. Therefore, our work has strong research significance.

Compared with the objective image quality assessment (IQA) methods of evaluating NSIs only, such as BRISQUE [12], NFERM [5], NIQE [13], HOSA [14] and SNP-NIQE [15], or IQA methods of evaluating SCIs only, such as SVQI [16], ESIM [17], BQMS [18], UCA [19], and ASIQE [19], the objective IQA method for comprehensively evaluating the above two different types of images is still in the initial stage of research. On the one hand, the DPQAD database only includes the distorted images on different screens, so only a no-reference quality evaluation method that does not require reference information can be designed. On the other hand, the monotonicity of the forecast is far from satisfactory.

In order to overcome the above shortcomings, we design a novel no-referenced (NR) quality assessment method of display products (NQMDP), considering that the authors of [9,20] have explained that complexity, contrast, sharpness, brightness, colorfulness, and naturalness are critical to image quality, so we extracted 27 image features based on the above six key factors that affect image quality and then learned the above features through SVR to infer the overall quality score of a given image. Moreover, because this method does not require any reference information, it is very practical in many practical application scenarios.

The rest of this paper is as follows. In Section 2, we introduce a subjective quality assessment method of mutual supervision, and then we apply it to the database that we established. Section 3 describes the novel IQA metric we designed. In Section 4, we discuss the experimental setup, results, and analysis. Finally, the conclusion is given in Section 5.

## 2. Subjective Quality Assessment of Display Products

In this section, we first introduce a subjective quality evaluation method of mutual supervision, then we describe the overall scheme and specific details of the implementation of subjective quality evaluation, and finally we analyze the results of the subjective quality evaluation experiment, which proves that the mutual supervision method has obvious reliability.

### 2.1. Mutually Supervised Subjective Evaluation

In the subjective quality evaluation, the single stimulation (SS) method, the double stimulation (DS) method, and the stimulation comparison (SC) method are the three most commonly used subjective scoring methods in the literature. Because we can only obtain the screen image by distorting the screen and cannot obtain the original image without distortion, the DS method cannot be used in this paper. However, the SS method and SC method have their own shortcomings. Specifically, the single stimulus method divides the subjective score into five levels, which are respectively marked as “excellent”, “good”, “fair”, “poor”, and “bad’, and are represented by the numbers from 5 to 1, as listed in Table 1.

It can be clearly seen that the single stimulation method has the advantages of being easy to implement and reflecting the direct opinions of human observers. Therefore, as reported in the standard of ITU-R BT.500 [21], it is the most widely used subjective assessment method. However, its shortcomings are also obvious: there are some differences in the five-level evaluation criteria (e.g., two images separately with quality of 4.1 and 4.3 are both scored as 4 and thus cannot be distinguished), this makes the final results very likely to be unreasonable.

Compared with the single stimulation method, the stimulation comparison method is mainly used for comparing the differences of quality between two systems (such as signals, images, and devices) [22]. Table 2 lists the principle of scoring by the SC method.

In consideration of the possibility that human observers cannot distinguish which image has the better quality, we add a new option “0” on the aforementioned SC method to make the popular SC method more robust. Generally speaking, the SC method is more accurate than the SS method since the SC method can avoid the interference such as the changes of picture scenes. Unfortunately, the SC method can only provide the qualitative results (for example, which is better between the two images), but is unable to directly derive the quantitative results (namely the quality score of the image).

**Figure 1:**
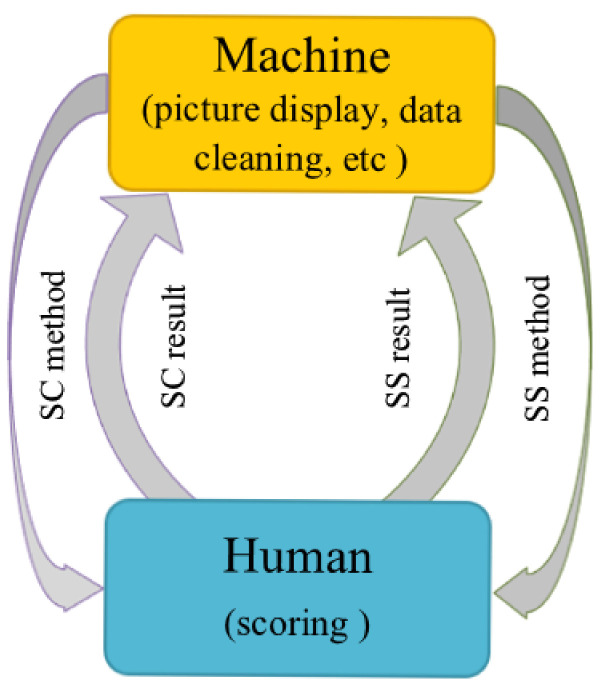
Symbolic diagram of the proposed BMS method.

Because the two commonly used subjective evaluation methods have some shortcomings, in order to solve the above problems and further improve the reliability of the subjective scoring results, we combine the above two methods to make them supervise each other, and propose a novel subjective quality evaluation method. We dubbed the newly proposed subjective evaluation method “Baseball-like Mutually-Supervised method” as BMS method because the whole process constitutes a baseball shape, as displayed in Figure 1.

The whole process can be classified into the following three steps: (1) Implementing the SS method to derive the quantitative results. (2) Implementing the SC method to derive the qualitative results. (3) Comparing the aforesaid qualitative and quantitative results to remove unreliable subjective evaluation scores, based on the mutual supervision strategy, and using the SS results as the final MOSs.

### 2.2. Protocol of Subjective Quality Assessment Experiment

In order to investigate the quality evaluation of the display products’ screen images, first of all, we prepare three display products with different screen parameters, namely, BOE RGBW screen (5.824’/1080*2244), BOE RGBW screen (5.824’/1080*2244), and Real RGB screen (5.9’/1080*1920). Table 3 lists the specific parameters of the three different types of display screens. These three display screens we use are all standard dynamic range devices, and the screen images displayed are all RGB color images. Therefore, the following experiments in this paper are carried out only in the RGB color space, and other color spaces are not discussed.

Second, we collect a total of 50 pictures of resolution 1080*1920, which can be divided into three categories and eleven sub-categories as follows. (1) Characters category: Person, Animation, Games, etc. (2) Scenery category: Urban scenery, Architecture, Pastoral scenery, etc. (3) Others: Webpage, WeChat, Map, etc. Then, we invite 57 human observers who have never encountered expert training, and then refer to the standards in [23] and design the following four steps: First, establishing the experimental environment in terms of the standard. Second, training the invited non-professional observers. Third, scoring the pictures by the invited observers. Fourth, removing the outliers. Reasonable scoring of images contains many implementation details. Next, we will explain the above four steps in detail.

A. Experimental Environment Establishing

**Figure 2:**
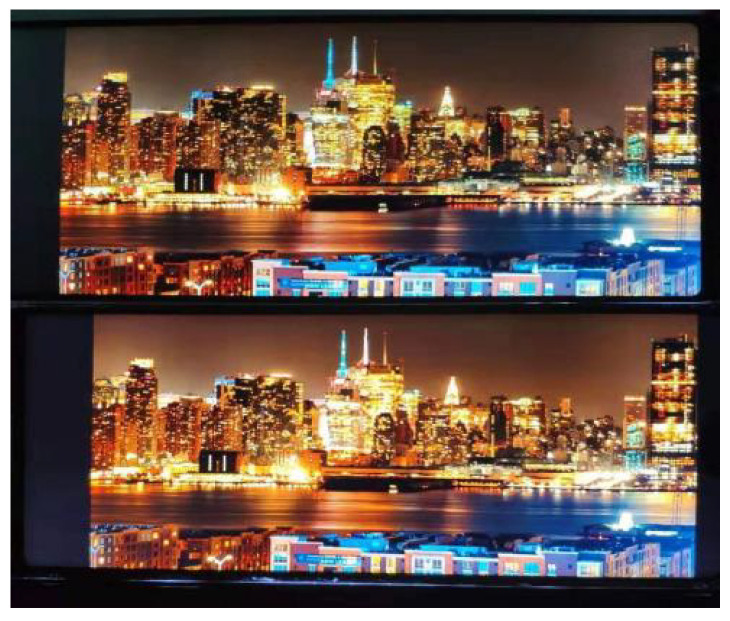
Experimental mobile phone screen.

1. We fix the two mobile phone screens to the background cardboard and keep them as the same as possible, as shown in Figure 2;
2. During this test, the ambient light condition is sufficient and steady. The light condition is kept the same in the whole screen, for avoiding the impacts caused by the environment;
3. Under the condition that the backlight current is set to 30 mA and the power consumption of the two products is kept the same, we adjust the color temperature of RGBW to be close to that of RGB;
4. The participants are kept at a distance of 30 cm from the phone screen and located in front of the screen to prevent the results from being affected by side-looking.

B. Observers Training

Appropriately training participants by informing them the scoring rules and basis, and making them focus on the visual quality such as display brightness, clarity, color saturation, etc.

C. Scoring Process

1. Conducting the SS method by asking the observers to score the pictures displayed on screen A and B. The time during assessing each picture is 20 s. The range of score is from 1 to 5;
2. Conducting the SC method by asking the observers to compare the same picture displayed on two different screens (A and B). The observers are requested to decide which screen has better display effect or whether the two screens have equivalent display effect. The time during assessing each picture is 20 s as well;
3. Each participant observes the same number of pictures at the same time before gives their subjective scores.

D. Data Cleaning

In the subjective evaluation experiment, the participants’ preference, emotion, and knowledge background will influence the evaluation results. Therefore, it is necessary to clean those abnormal data in advance, for improving the reliability of results.

1. Cross-test-based data cleaning: Abnormal data were cleaned based on the comparison of SS’s results and SC’s results. If the score of SS method is opposite to that of SC method, the relevant data will be discarded.
2. Cross-content-based data cleaning: Considering the different contents of pictures, this paper independently analyzes each picture and cleans the outliers in each picture. According to the results of SS method, the average score and 95% confidence interval of each picture are derived. Unreasonable scores outside the interval are deleted according to the method in [24].
3. Cross-evaluator-based data cleaning: After completing the above two data cleaning methods, it is required to exclude the subjective scores of special testers. When more than 20% of data provided by a single participant are discarded, that participant is considered to be “careless” during the test or his/her hobbies deviate from the public. If so, all of the data of the participant are deleted.

### 2.3. Subjective Assessment Experimental Results

After data cleaning, we take the mean of all valid subjective scores of each picture as the screen display quality score for one certain picture. To demonstrate the effectiveness of our BMS method, we invited a senior expert to assess the picture quality. As is well known, the subjective score cannot be a general linear mapping, so logistic regression is used to perform nonlinear mapping of the quality score of each image. Then we use the mapped score and the score given by the expert to calculate the linear correlation coefficient (LCC) and root mean square error (RMSE), as reported in Table 4. The specific calculation equations are shown in Equations (1) and (2).

(1) $$LCC=\frac{1}{num-1}\sum_{i=1}^{num}\left[\frac{\left(sm_{i}-\bar{sm}\right)}{\sigma_{sm}}\right]\left[\frac{\left(se_{i}-\bar{se}\right)}{\sigma_{se}}\right],$$

(2) $$RMSE={\left[\frac{1}{num}\sum_{i=1}^{num}{\left(sm_{i}-se_{i}\right)}^{2}\right]}^{\frac{1}{2}},$$

where num indicates the number of scores, sm and se represent the score after the mapping and the score given by the expert, respectively. $\bar{sm}$ and $\bar{se}$ represent their mean values, and $\sigma_{sm}$ and $\sigma_{se}$ represent their standard deviations. Note that a value close to 1 in LCC and close to 0 in RMSE indicates the higher the correlation between the two. As seen, our proposed BMS is more reliable than the SS method.

## 3. Objective Quality Assessment of Display Products

**Figure 3:**
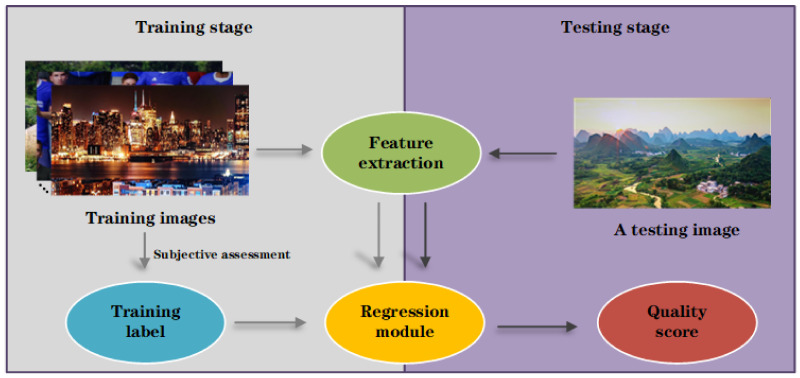
The algorithm framework of NQMDP.

In this section, we propose a novel no-reference quality assessment method of display products, which is called NQMDP. Figure 3 shows our method’s algorithm framework. Specifically, the framework is divided into two stages: (1) Training stage: Send the extracted features of the training images to the regression module, so that the quality score keeps getting closer to the training label. (2) Testing stage: Send the extracted features of a test image to the regression module, and output its quality score through the regression module. It is obvious that the two main parts of the algorithm framework are feature extraction and regression module.

In this section, the six different aspects of feature extraction are in detailed description first: complexity, contrast, sharpness, brightness, colorfulness, and naturalness. These quality perception features that affect image quality are widely applied in NR-IQA [25,26,27] and have yielded good results. Second, the integrated SVR technology in the regression module is mainly explained.

### 3.1. Feature Extraction

Our proposed method is composed of six types of features. The first type of complexity features are related to the effects of gaze direction and spatial masking [11]. The autoregressive (AR) model and bilateral filter (BL) have been successfully applied to estimate the image complexity [28,29], but both AR and BL have their own limitations. The autoregressive (AR) model works very well on the textured regions, but it does not perform well near the image edges where the ringing artifacts are introduced. The bilateral filter (BL) has edge-preserving capability, but it does not perform well on the textured regions. Following the previous studies [8], in order to obtain the better performance on the AR and BL filters, we combine the above two filters systematically:

(3) $${\hat{T}}_{i}=\frac{J^{n}\left(x_{i}\right)\hat{\alpha }+\tau J^{n}\left(x_{i}\right)\beta }{1+\tau }.$$

By designing this hybrid filter, the advantages of AR and BL filters are combined, and the good compromise between AR and BL filters is embodied, where *i* is the index of a query pixel, ${\hat{T}}_{i}$ is the value of a pixel at location $x_{i}$, the *n* neighboring pixels of $x_{i}$ that make up $J^{n}\left(x_{i}\right)$, $\hat{\alpha }$ and β are coefficients produced by AR and BL filtering, and τ is the weight parameter used to adjust the response intensity of AR and BL models. Here, we set the weight value at τ=9. Although a simple linear weighting function with fixed weights is currently used, an adaptive weighting scheme may be better and will be investigated in future work. Next, we calculate the residual error map $\Delta T_{i}=T_{i}-{\hat{T}}_{i}$, where the larger absolute value corresponds to the highly complex texture region and the smaller absolute value corresponds to the less complex or smooth region. Then, the entropy of the residual error map $\varepsilon_{o}$ is defined as

(4) $$\varepsilon_{o}=-\int_{\lambda }P_{\lambda }logP_{\lambda }d\lambda ,$$

where $P_{\lambda }$ is the probability density of the λ-th grayscale in the error map $\varepsilon_{o}$. Previous neuropsychological studies [30,31] have shown that the human visual system has a selective mechanism to narrow ranges of spatial frequency and direction, and this research has been widely applied in multi-scale visual modeling. Therefore, we also measure the image complexity after reduced resolution and express the reduced resolution image complexity as $\varepsilon_{d}$. Specifically, the image complexity is measured by applying a 16 × 16 square moving low-pass filter and sub-sampling in steps of 16 pixels along each main direction. Therefore, the first type of complexity features are described as $\varepsilon =\{\varepsilon_{o},\varepsilon_{d}\}$.

The second type of contrast features estimate the perceived local contrast of the image. We apply Gaussian second-order derivative filters to separate the image, and then calculate the contrast energy on three channels:

(5) $$E_{c}=\frac{\omega \cdot \gamma (k_{c})}{\gamma (k_{c})+\omega \cdot \vartheta }-\Phi_{c},$$

where $\gamma \left(k_{c}\right)={\left({\left(k_{t}*c_{x}\right)}^{2}+{\left(k_{t}*c_{y}\right)}^{2}\right)}^{1/2}$; $c=\{c^{1},c^{2},c^{3}\}$ are three channels of *k*, respectively, where $c^{1}=0.299R+0.587G+0.114B$, $c^{2}=0.5\left(R+G\right)-B$ and $c^{3}=R-G$. For parameters, $\omega =max[\gamma \left(k_{c}\right)]$, ϑ governs the contrast gain, and $\Phi_{c}$ is applied to constrain the noise with threshold. $c_{x}$ and $c_{y}$ stand for horizontal and vertical second-order derivatives of Gaussian function [32]. Therefore, the contrast features are $E_{c}$ = $\left\{E_{c_{1}},E_{c_{2}},E_{c_{3}}\right\}$.

In general, sharpness features are more susceptible to local variations, which means that the fine details of a image can usually be distinguished in sharp areas (such as edges and object boundaries). Therefore, the third type of image features used by this method is the sharpness features. We first employ 9/7 DWT filters to decompose the grayscale image into three levels, namely, $\left\{LH_{n},LH_{n},LH_{n}|n=1,2,3\right\}$. Considering that high-sharp images usually contain more high-frequency details, we calculate the log-energy of each wavelet sub-band at each decomposition level:

(6) $$SE_{PQ_{n}}=log_{10}[1+\frac{1}{N_{n}}\sum_{i,j}PQ_{n}^{2}\left(i,j\right)],$$

where (i,j) stands for the pixel index; PQ is LH, HL, and HH respectively; and $N_{n}$ is the total number of DWT coefficients at the level n. The log energy at each decomposition level is computed by

(7) $$SE_{n}=\frac{\left(SE_{LH_{n}}+SE_{HL_{n}}\right)/2+a\cdot SE_{HH_{n}}}{1+a},$$

where *a* is set to 4, so as to impose larger weights on the HH sub-bands. Note that the experimental results show that adding the first level feature does not lead to performance improvement. Therefore, to improve efficiency without affecting performance, we merely consider the second and third level features that involve clearer details [33]. Therefore, the sharpness features are SE = $\left\{SE_{2},SE_{3}\right\}$.

The brightness function is the fourth type of image features in our method, because the appropriate brightness can make the image have a wider dynamic range, so as to better help the image display more details. The sample mean of image *I* is used to represent the first feature of image brightness:

(8) $$B_{o}=e\left(I\right)=\frac{1}{N}\sum_{n=1}^{N}I\left(n\right).$$

This feature captures brightness shifts due to errors in improper postprocessing technologies. Generally speaking, the image details would disappear as the intensity of the brightness changes continuously. Next, we infer whether the image has appropriate brightness by measuring the information entropy of the image with brightness change. When more multiplier indexes are used, the calculation speed becomes slower, but the performance becomes better. Therefore, in order to achieve a good balance between performance and efficiency, the NQMDP model we proposed uses the following six entropy values to measure image brightness: $\left\{B_{p1},B_{p2},...,B_{p6}\right\}$, where *p* is denoted as {q,(1/q)∣q=3.5,5.5,7.5} [34]. Therefore, the brightness features are *B* = $\left\{B_{o},B_{p1},B_{p2},B_{p3},B_{p4},B_{p5},B_{p6}\right\}$.

The fifth type of colorfulness features is based on the fact that color information can provide a wider dynamic range, thus displaying more details and information relative to grayscale images. We introduce color saturation to quantify the color of the image. Specifically, after transforming an image into the HSV color space, we merely need to calculate the global mean value of the saturation channel:

(9) $$C^{⋆}=\frac{1}{N}\sum_{n=1}^{N}TF_{RGB\to S}\left[I\left(n\right)\right],$$

where $TF_{RGB\to S}$ stands for a transformation function to convert a RGB type image into the color saturation channel, and *N* represents the number of pixels in image *I*. As the previous color appearance models can only be effective for simple blocks on a uniform background, in order to estimate the colorfulness of the entire image from the perspective of human visual perception, Hasler et al. proposed the image colorfulness metric [35] which is highly correlated with human perception through key feature extraction and psychophysical category scaling experiments. Specifically, we extract firstly the four key features, including the mean and variance of the c2 and c3 channels ($\mu_{c2}$, $\sigma_{c2}^{2}$, $\mu_{c3}$ and $\sigma_{c3}^{2}$ ). c2 and c3 are defined in Equation (7). Then, the metric is defined by

(10) $$C^{*}=\eta \cdot \sqrt{\mu_{c2}^{2}+\mu_{c3}^{2}}+\sqrt{\sigma_{c2}^{2}+\sigma_{c3}^{2}},$$

where η is set to 0.3, which is a parameter that corrects the relative importance. Therefore, the colorfulness features are *C* = $\left\{C^{⋆},C^{*}\right\}$.

We consider the sixth type of naturalness features, as they represent some commonness of majority NSIs. If a image violates these commonness, it means that the image looks unnatural, resulting in low visual quality. First, an image is preprocessed by local mean removal and divisive normalization. Next, we estimate the coefficients of the preprocessed image with the zero-mean generalized Gaussian distribution, and take $\mu_{n}$ that controls the shape of the distribution and the variance $\sigma_{n}^{2}$ of the distribution as two features of naturalness. The third feature of naturalness comes from DCP prior [36], which shows that in most non-sky areas, at least one color channel tends to zero:

(11) $$NL_{dark}\left(n\right)=\min_{c\in R,G,B}I_{c}\left(n\right),$$

where *c* = {R,G,B} represents the RGB channel. We merely calculate the overall mean of the dark channel $NL_{dark}$ as the naturalness measurement value $NL_{d}$. Therefore, the features of naturalness are defined as NL = $\left\{\mu_{n},\sigma_{n}^{2},NL_{d}\right\}$.

For naturalness, another statistical feature we extracted is patch-based [37]. Specifically, we test the degradation information of the image structure in the following way. Given an image I, we indicate $\mu_{I}$,$\sigma_{I}$ and $\tilde{\sigma_{I}}$ as local mean and variance maps:

(12) $$\mu_{I}=\sum_{k=1}^{K}\omega_{k}I_{k}$$

(13) $$\sigma_{I}={\left[\sum_{k=1}^{K}\omega_{k}{\left(I_{k}-\mu_{I}\right)}^{2}\right]}^{\frac{1}{2}}$$

(14) $$\tilde{\sigma_{I}}={\left[\sum_{k=1}^{K}{\left(I_{k}-\mu_{I}\right)}^{2}\right]}^{\frac{1}{2}},$$

where $\omega =\{\omega_{k}|k=1,2,...,K\}$ is a normalized Gaussian window. The structural degradation is then measured by

(15) $$SD_{\mu }\left(I\right)=\frac{1}{N}\sum \frac{\sigma_{\left(\mu_{I},I\right)}+\zeta }{\sigma_{\left(\mu_{I}\right)}\sigma_{I}+\zeta }$$

(16) $$SD_{\sigma }\left(I\right)=\frac{1}{N}\sum \frac{\sigma_{\left(\sigma_{I},\tilde{\sigma_{I}}\right)}+\zeta }{\sigma_{\left(\sigma_{I}\right)}\sigma_{\left(\tilde{\sigma_{I}}\right)}+\zeta },$$

where *N* is the number of pixels in image *I*; ζ is an additional fixed positive constant. Considering that the screen image of the display product contains both image content and text content, we plan to use a Gaussian window function with size of 11 × 11 and standard deviation of 1.5 to capture the macroscopic structure information of the image, while using a Gaussian window function with size of 3 × 3 and unit standard deviation to capture the microscopic detailed information of the image [38]. That is to say, we use the above two Gaussian window functions to calculate Equations (12)–(16). In general, when block-based compression distortion is introduced into an image, the interior of the coded block is usually smoothed by the zeroing the of the high-frequency block DCT coefficients, and block artifacts are introduced along the edges of the block. Therefore, when extracting structural degradation information, we treat the interior and edges of blocks in different ways, unlike when dealing with other types of distortion (such as noise and blur) [39].

This analysis produces eight structural degradation features, denoted as $SD_{\left(u,v,w\right)}$, where u={μ,σ} represents the information type, v={3,11} represents the kernel size, and w={in,ed} represents block interiors and edges, respectively. From the previous study [9], we know that there is an obvious near-linear relationship between the undistorted images’ complexity characteristics and structural degradation characteristics. In other words, the larger the image complexity is, the smaller the image degradation information is. In order to predict the possibility of visual distortion in the screen image of the displayed product, we try to fit the linear regression model:

(17) $$\varepsilon_{o}\left(I\right)={\left[\begin{array}{c} X_{\left(u,v,w\right)}\\ Y_{\left(u,v,w\right)} \end{array}\right]}^{T}[\begin{array}{c} SD_{\left((u,v,w\right)}\left(I\right)\\ 1 \end{array}],$$

where $\left[X_{\left(u,v,w\right)},Y_{\left(u,v,w\right)}\right]$ indicates one of eight parameter pairs corresponding to (u,v,w). We use the least square method to estimate these parameters.

**Figure 4:**
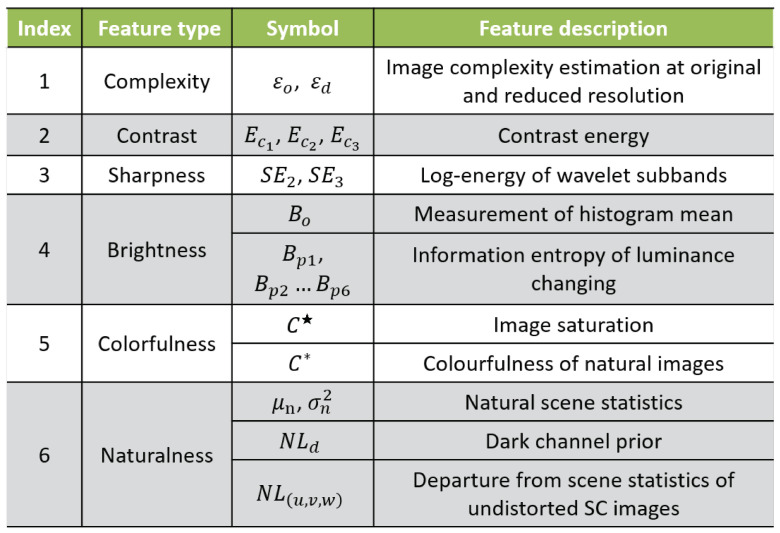
Summary of features for NQMDP.

According to the linear regression model above, we define $NL_{\left(u,v,w\right)}\left(I\right)=\varepsilon \left(I\right)-\left(X_{\left(u,v,w\right)}\cdot SD_{\left(u,v,w\right)}\left(I\right)+Y_{\left(u,v,w\right)}\right)$. When the image quality is higher, the value of $NL_{\left(u,v,w\right)}\left(I\right)$ is closer to 0. We take the scene statistical departure feature $NL_{\left(u,v,w\right)}$ as the second feature of naturalness, where u={μ,σ}, v={3,11}, and w={in,ed}. We summarize the above six different types of features in Figure 4.

### 3.2. Regression Module

Next, we should effectively combine the 27 features of the six aspects above to provide prediction scores of the visual quality of the screen images on the display products. To this end, we deployed a regression module using support vector regression (SVR) [12,40,41] to convert features into quality scores. Specifically, we use the LibSVM package to implement the SVR, where the kernel function of the SVR uses the radial basis function (RBF) kernel [42]. In order to verify whether our model is effective, we divide 80% data of the DPQAD database for training the SVR model, and the remaining 20% for testing the SVR model. Then we iteratively calculate the average result of the 1000-time leave-one-out cross-validation experiment as the final result of the experiment. As the training and testing data only contain limited scenes and distortions, it is difficult to guarantee that it still maintains outstanding performance in a wider range of scenes and distortions. In order to solve the above problem, we adopt the integrating SVR technique to improve the robustness of the proposed model. Specifically, in order to obtain stable and accurate prediction quality scores for our model, we try to train and test the above data using three SVR models with different parameters. The different parameters refer to the penalty coefficient of the loss function in the SVR model. Generally, the penalty coefficient in the SVR model is set to 1 by default. In our experiment, the penalty coefficient of the three SVR models is set to 5, 20 and 100 respectively. Then the quality scores predicted by the three SVR models are averaged and used as the final prediction score of our model. In future work, we plan to explore more complex and efficient learning methods.

## 4. Experiments and Discussion

In this section, we will demonstrate the performance of our method(NQMDP) on the quality assessment database of display products (DPQAD). The DPQAD includes three categories: character category, scene category, and other categories, with a total of 150 screen images displayed on the product. The subjective quality score of each image in the database is evaluated by 57 non-professional volunteers. More details of subjective evaluation are described in Section 2 and will not be repeated here.

In order to better verify the obvious superiority of our proposed NQMDP model, we compared NQMDP with ten state-of-the-art relevant IQA models, which include five classes: (1) evaluation methods for NSIs including NFERM [5] and HOSA [14]; (2) evaluation methods for image sharpness including ARISMC [43] and HVS [44]; (3) evaluation methods for image contrast including NIQMC [8] and BIQME [6]; (4) evaluation methods without opinion scores including NIQE [13] and SNP-NIQE [15]; (5) evaluation methods for SCIs including BQMS [18] and ASIQE [19]. In order to fairly compare the performance of all the above algorithms, we used the five metrics recommended by the video quality expert group [45]. That is, Kendall’s rank correlation coeffcient (KRCC) and Spearman rank correlation coeffcient (SRCC) are used to evaluate and prediction monotonicity, Pearson linear correlation coeffcient (PLCC) is used to evaluate the prediction accuracy, and mean absolute error (MAE) and root mean-squared error (RMSE) are used to evaluate the prediction consistency. Generally speaking, the predicted values of PLCC, SRCC, and KRCC are closer to 1, and the predicted values of MAE and RMSE are closer to 0, indicating that the prediction performance of the model is better. As is known to all, subjective score cannot be a general linear mapping, so we use logistic regression to carry out a nonlinear mapping of the predicted quality score of each image:

(18) $$F\left(s\right)=a_{1}\left(\frac{1}{2}-\frac{1}{1+e^{a_{2}\left(s-a_{3}\right)}}\right)+a_{4}s+a_{5},$$

where *s* is the predicted quality score, F(s) is the quality score obtained after logistic regression, and $a_{1}–a_{5}$ are the five parameters determined in the curve fitting process.

**Figure 5:**
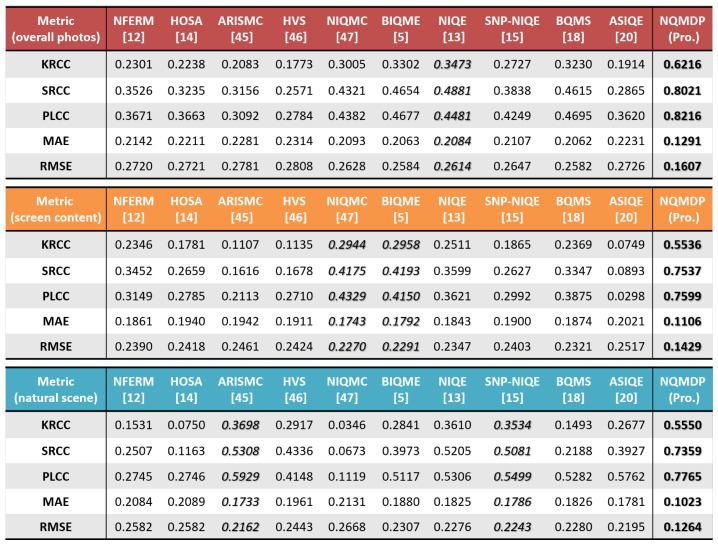
Comparisons of 10 state-of-the-art relevant algorithms ON DPQAD (150 images).

First, we validate the performance of our proposed NQMDP based on 80% data for training and the rest data for testing. Results of our method are represented as the average value on 1000 iterations, as reported in Figure 5. The best value in Figure 5 is highlighted in bold, and the second best value is in italic. It can be seen that the performance in terms of prediction monotonicity, prediction accuracy, and prediction consistency has reached the best performance, and the prediction monotonicity and prediction accuracy both exceed 80%. The other ten blind quality methods are also examined, and their results are listed in Figure 5. Among them, NIQE has derived the highest performance among the ten comparison methods (italic to highlight), with its KRCC, SRCC, PLCC, MAE, and RMSE being 0.3473, 0.4881, 0.4481, 0.2084, and 0.2614, respectively. In comparison, it is clear that our method is better than the second-place NIQE, having achieved the relative performance gain of 78.98%, 64.33%, and 83.35% in terms of KRCC, SRCC, and PLCC, respectively. Relative error reduction of 38.05% and 38.52% are achieved in terms of MAE and RMSE, respectively. Furthermore, we check the performance of our method and ten state-of-the-art comparison methods on 93 SCIs and 57 NSIs. We provide their results in the last two tables in Figure 5. As seen, our NQMDP has acquired the highest performance on both two types of images, achieving 0.7599 and 0.7765 in light of PLCC. BIQME and NIQMC have derived superior performance on SCIs, with their PLCC values being 0.4150 and 0.4329. However, the two methods perform poorly on NSIs. By contrast, SNP-NIQE and ARISMC have attained good performance on NSIs, with their PLCC values being 0.5499 and 0.5929, but fail in SCIs. It can be concluded that our NQMDP method is significantly better than the other 10 methods on both NSIs and SCIs.

**Figure 6:**
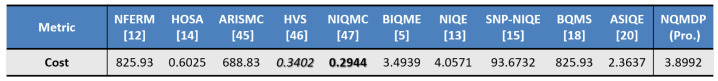
The comparison between NQMDP model and other 10 models in execution efficiency (second/image).

In addition to the above five metrics, execution efficiency is also an important indicator. We are using a win10 system server, which has an Intel(R) Xeon(R) CPU E5-2620 v4 at 2.10 GHz with 192.00 GB of RAM. In our experiment, we use MATLAB R2014a to test our proposed model and the other 10 comparison models. As can be seen from Figure 6, NIQMC gets the optimal value highlighted in bold, HVS gets the suboptimal value in italic. In addition, the average time for NQMDP model to predict the quality score of each image is less than four seconds, far exceeding the execution efficiency of most models. This is because the manual features we designed are not only very effective, but also has low computational complexity. Although HOSA, HVS, ASIQE, and BIQME consume less time than the NQMDP model, the prediction performance of these four methods is much lower than that of NQMDP. In addition, compared with the second-place NIQE model, the NQMDP model we proposed has improved its execution efficiency by 3.89%.

**Figure 7:**
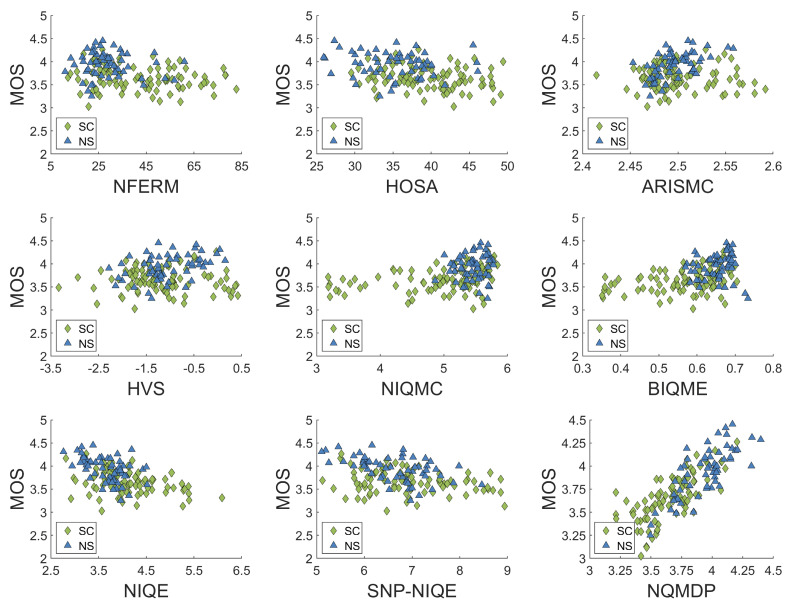
Scatter plots of MOS versus NFERM [5], HOSA [14], ARISMC [43], HVS [44], NIQMC [8], BIQME [6], NIQE [13], SNP−NIQE [15], and our NQMDP on DPQAD database.

Afterwards, in order to intuitively show the performance of NQMDP model from the qualitative perspective, we drew the scatter plots between objective quality prediction scores and subjective evaluation quality scores obtained by the above 10 comparison models and NQMDP model, as shown in Figure 7. In each scatter plot, we use different color markers to represent different types of images. Among them, the green diamond represents the SCIs and the blue triangle represents the NSIs. By analyzing all the scatter plots above, we can easily get two conclusions: (1) Compared with other 10 IQA models, our NQMDP model has higher monotonicity and linearity. Specifically, the scatter plot of the NQMDP model is more slender than the scatter plot of the NIQE model (second place). (2) We expect to design an excellent quality evaluation model that can effectively evaluate both NSIs and SCIs. Specifically, ARISMC, ASIQE, HOSA, HVS, and SNP−NIQE cannot evaluate SCIs well; HOSA, NFERM, and NIQMC cannot evaluate NSIs well. It can be clearly observed that only the NQMDP model we proposed can effectively evaluate the above two different types of images and has strong robustness.

**Figure 8:**
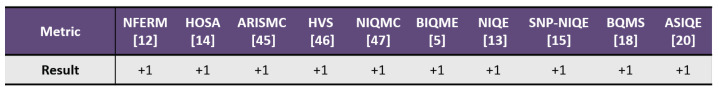
The comparison between NQMDP model and other 10 models in F-test (statistical significance).

Finally, we use the F-test to analyze the statistical significance of the NQMDP model. Specifically, first calculate the residuals of the prediction scores of the two objective evaluation methods and the corresponding subjective evaluation scores, and then use the ratio of the residual variances as the F value. In the F-test experiment, we set the judgment threshold F-critical to 0.05 (determined by the confidence level). By comparing F-critical with the F values calculated by the NQMDP model and other methods, the reasonable statistical judgment can be made on the NQMDP model. The statistical significance results are shown in Figure 8, where the symbol “0” indicates that there is no significant difference between the two methods, “−1” indicates that our method is statistically inferior to the other method, and “+1” indicates our method is statistically superior to the other method. By observing the results in Figure 8, it is easily can be seen that our proposed NQMDP model has a significant performance improvement in terms of statistical significance. It is worth mentioning that the confidence of the results in Figure 8 is 95%.

## 5. Conclusions

In this paper, we have studied an important but less researched direction, which is subjective and objective quality assessment of display products. In recent years, the time that humans spend on display products has greatly increased, and there has been an increasing demand for a high-quality visual experience of display screens. Therefore, we have conducted in-depth research on the subjective and objective quality assessment of display products, so as to guide the reasonable screen parameters settings of the display products. Note that we have made two main contributions. First, as there are few previous studies in this area, we have established a new screen image database of display products. In order to improve the reliability of subjective scoring results, we have adopted a subjective quality evaluation method combining a single stimulation method and stimulation comparison method in an innovative manner. Second, in order to automatically and objectively evaluate the screen images of the display products, we designed a novel no-reference quality assessment framework for the display products. The framework appropriately integrates six key factors that affect image quality, and learns the above features by using SVR machine learning method, so as to infer the final quality score of a given image. We have conducted extensive experiments on the above-mentioned database to compare our method with 10 state-of-the-art objective evaluation methods. The experimental results prove that our method is superior to its competitors in almost every aspect. It is worth mentioning that there are huge differences between the NSIs and the SCIs, and the screen images of display products contain both of the above types of images. We have taken this into consideration when designing objective quality evaluation method. Therefore, our method shows excellent performance whether evaluating NSIs or evaluating SCIs.

In the future work, our plan is divided into two parts: (1) Expand the database of display products’ screen images, and increase the number of different types and different content of display products’ screen images. (2) Design more effective image manual features, reduce the number of manual features used, and improve the execution efficiency of the method on the premise of further improving the performance of the objective quality evaluation method.

## Figures and Tables

**Table 1:** Principle of scoring by single stimulation method.

Score	Quality	Impairment
5	Excellent	Imperceptible
4	Good	Perceptible, but not annoying
3	Fair	Slightly annoying
2	Poor	Annoying
1	Bad	Very annoying

**Table 2:** Principle of scoring by stimulation comparison method.

Score	Condition	Explanation
+1	A > B	A is better than B
0	A = B	A is equivalent to B
−1	A < B	A is worse than B

**Table 3:** Detailed parameters of the three display screens.

	Resolution	Luminance Peak	Contrast	Flicker	Color Temperature	Gamma
BOE RGBW screen	1080*2244	625.3	1125.4	0.5	7688.9	2.12
BOE RGBW screen	1080*2244	708.8	1228.4	0.3	6503.1	2.17
Real RGB screen	1080*1920	341.8	1393.9	0.5	7581.2	2.14

**Table 4:** Principle of scoring by stimulation comparison method.

Index	RGB		RGBW	
	SS	BMS	SS	BMS
LCC	0.9835	0.9866	0.9888	0.9933
RMSE	0.0381	0.0320	0.0311	0.0234

## Data Availability

The experiment uses an internal data set. The data presented in this study are available on request from the corresponding author.
